# Association between genetic polymorphisms of MMP8 and the risk of steroid-induced osteonecrosis of the femoral head in the population of northern China

**DOI:** 10.1097/MD.0000000000004794

**Published:** 2016-09-16

**Authors:** Jieli Du, Tianbo Jin, Yuju Cao, Junyu Chen, Yongchang Guo, Mingqi Sun, Jian Li, Xiyang Zhang, Guoqiang Wang, Jianzhong Wang

**Affiliations:** a Inner Mongolia Medical University, Hohhot, Inner Mongolia; b The College of Life Sciences, Northwest University; c National Engineering Research Center for Miniaturized Detection Systems, Xi’an, Shanxi; d Zhengzhou TCM Traumatology Hospital, Zhengzhou, Henan; e Department of Orthopedics and Traumatology, The 2th Affiliated Hospital of Inner Mongolia University, Hohhot, Inner Mongolia, China.

**Keywords:** association study, MMP8, single-nucleotide polymorphism, steroid-induced osteonecrosis of the femoral head

## Abstract

**Background:**

Steroid-induced osteonecrosis of the femoral head (ONFH) is the most common clinical nontraumatic ONFH. Once ONFH occurs, it seriously reduces patients’ quality of life. The matrix metalloproteinase/tissue inhibitor of metalloproteinase (MMP/TIMP) system was found to play a significant role in the development of ONFH. The aim of this study was to identify the associations between 7 genes selected from the MMP/TIMP system and steroid-induced ONFH.

**Methods:**

We genotyped 34 single-nucleotide polymorphisms (SNPs) of 7 genes selected from the MMP/TIMP system in a case–control study with 285 cases of steroid-induced ONFH and 308 healthy controls. Odds ratios (ORs) and 95% confidence intervals (CIs) were estimated using the chi-squared test, genetic model analysis, haplotype analysis, and stratification analysis.

**Results:**

We found that the minor alleles of rs1940475 and rs11225395 in *MMP8* were associated with a 1.32-fold increased risk of steroid-induced ONFH in the allelic model analysis (*P* = 0.021 and 0.022, respectively). In the genetic model analysis, we found that rs3740938, rs2012390, rs1940475, and rs11225395 were associated with an increased risk of steroid-induced ONFH. In further stratification analysis, rs3740938 and rs2012390 displayed a significantly increased risk of steroid-induced ONFH in females under the dominant (rs3740938, OR = 2.69, 95% CI: 1.50–4.83, *P* = 0.001; rs2012390, OR = 2.30, 95% CI: 1.31–4.03, *P* = 0.012) and additive (rs3740938, OR = 2.02, 95% CI: 1.24–3.29, *P* = 0.010; rs2012390, OR = 1.77, 95% CI: 1.12–2.80, *P* = 0.047) models. In addition, haplotype “AGTCA” of *MMP8* was found to be associated with a 1.40-fold increased risk of steroid-induced ONFH (95% CI: 1.04–1.88, *P* = 0.025).

**Conclusion:**

Our results verify that genetic variants of *MMP8* contribute to steroid-induced ONFH susceptibility in the population of northern China. In addition, we found that gender differences might interact with MMP8 polymorphisms to contribute to the overall susceptibility to steroid-induced ONFH.

## 1 Introduction

Osteonecrosis of the femoral head (ONFH) is a common disease encountered in clinical work. With the wide use of glucocorticoids for the treatment of rheumatic, autoimmune, and hematopoietic-system diseases, steroid-induced ONFH has become the most common type of clinical nontraumatic ONFH. ONFH occurs in 39% of patients with severe acute respiratory syndrome within a few months of treatment with glucocorticoid therapy.^[1]^ Once the ONFH occurs, it seriously reduces the patients’ quality of life and is difficult to reverse. At present, there are several hypotheses about the causes of steroid-induced ONFH, including a hypercoagulable state,^[2]^ intraosseous pressure changes,^[3]^ fat embolism,^[4]^ bone-cell apoptosis,^[5,6]^ and circulatory impairment,^[7]^ but the exact pathogenesis is still unknown. Recently, associated studies revealed genetic polymorphisms of enzymes contributing to individual differences in steroid metabolism, steroid receptors, and transport proteins in patients with steroid-induced ONFH.^[8,9]^ All of these results suggest that genetic factors might play an important role in the development of steroid-induced ONFH.

Matrix metalloproteinases (MMPs), a family of zinc-dependent proteolytic enzymes, are expressed by stromal cells, infiltrating inflammatory cells, cancer cells, and osteoclasts. MMPs play central roles in morphogenesis, wound healing, tissue repair, and remodeling in response to injury.^[10]^ Tissue inhibitors of metalloproteinases (TIMPs), a family of natural inhibitors of both MMPs and pro-MMPs, inhibit the activities of MMPs by creating crystal structures in TIMP–MMP complexes.^[11,12]^ Previous studies^[13–15]^ reported that genetic variations of the MMP/TIMP system could cause the abnormal activation of osteoclasts, which might result in further ONFH. MMP-1 and MMP-3 are the key enzymes of collagen formation on the bone surface, which can promote the reabsorption of bone, leading to bone repair.^[16–18]^ MMP-2, -9, and -13 might act on osteoblast and osteoclast behavior and development in bone growth.^[19]^ MMP-1, -2, -7, and -9 are involved in the development of metastatic prostate cancer in bone.^[20]^ TIMP-1 and -2 can stimulate the bone-resorbing activity of osteoclasts by inhibiting the responsible MMPs, such as MMP-2 and -9, or the activation of the MAPK pathway.^[21]^ MMP-8 was expressed intensely in the process of osteoblast development into osteocytes.^[22]^

Recently, numerous studies have reported associations between genes and steroid-induced ONFH. However, there is little insight into the association between gene polymorphisms in the MMP/TIMP system and steroid-induced ONFH. In the present study, we selected 34 single-nucleotide polymorphisms (SNPs) of 7 genes from the MMP/TIMP system that have been previously reported to be associated with the risk of steroid-induced ONFH or of diseases with pathogenesis similar to that of steroid-induced ONFH. Our study aims to determine the relationship between genetic polymorphisms of the TIMP/MMP system and the risk of steroid-induced ONFH as well as to explore the interaction between the TIMP/MMP system and gender factors for the risk of steroid-induced ONFH.

## 2 Materials and methods

### 2.1 Study participants

A total of 285 patients with steroid-induced ONFH were recruited between September 2014 and January 2016 at Zhengzhou Traditional Chinese Medicine Traumatology Hospital. ONFH was diagnosed based on evidence of osteonecrosis on anteroposterior and frog-view X-rays of both hips and/or magnetic resonance imaging.^[23]^ Steroid-induced ONFH was defined by a history of a mean daily dose ≥16.6 mg or a high-dose steroid impulsion therapy for more than 1 week.^[24–26]^ We used detailed exclusion criteria: patients who did not meet the diagnostic criteria of steroid-induced ONFH or had traumatic ONFH, dislocation of the hip joint, or other hip diseases; patients who drank more than 400 mL ethanol per week; patients who had significant familial hereditary disease; and patients who did not agree to participate in the study.

Simultaneously, a total of 308 healthy, unrelated controls were recruited based on a medical examination at the Zhengzhou Traditional Chinese Medicine Traumatology Hospital. All of the chosen subjects were Han Chinese and lived in Zhengzhou city or the surrounding area. The controls did not have steroid-induced ONFH or other related diseases. Individuals with excessive use of corticosteroids, alcohol consumption, or significant familial hereditary disease were excluded.

Our study protocol adhered to the principles of the Declaration of Helsinki and was reviewed and approved by the Ethical Committee of Zhengzhou Traditional Chinese Medicine Traumatology Hospital. Informed consent was obtained from all candidate subjects.

### 2.2 SNPs selection and genotyping

All 34 SNPs had minor allele frequencies >5% in the HapMap Chinese Han Beijing population and were previously shown to be associated with the risk of steroid-induced ONFH or of diseases with pathogenesis similar to that of steroid-induced ONFH. Blood samples were collected in EDTA tubes and stored at −80 °C after centrifugation at 2000 rpm for 10 minutes. The GoldMag extraction method (GoldMag Co. Ltd, Xi’an, China) was used to extract genomic DNA from whole blood. The DNA quantity was evaluated by spectrometry (DU530UV/VIS spectrophotometer, Beckman Instruments, Fullerton, CA). The Sequenom MassARRAY Assay Design 3.0 Software was used to design a Multiplexed SNP MassEXTEND assay (Sequenom, Inc., San Diego, CA). A Sequenom MassARRAY RS1000 was used to perform the SNP genotyping according to the manufacturer's protocol.

### 2.3 Statistical analyses

All of the statistical analyses were performed with the SPSS 19.0 software for Windows (SPSS, Chicago, IL). Allele and genotype frequencies were obtained by direct counts. The genotype frequencies of each SNP among the control subjects were checked for Hardy–Weinberg equilibrium (HWE) before analysis. In the present study, all *P* values were 2-sided, and *P* ≤ 0.05 was considered to have statistical significance. The allele and genotype frequencies among the cases and controls were calculated by chi-squared test/Fisher exact test. Associations between the genotype polymorphisms and the risk of steroid-induced ONFH were evaluated under different genetic models (codominant, dominant, recessive, overdominant, and log-additive). Odds ratios (ORs) and 95% confidence intervals (CIs) were calculated using unconditional logistic regression analysis with an adjustment for gender and age. Then, we further analyzed the association between genotype and the risk of steroid-induced ONFH using each of the genetic models stratified by sex, and we tested the *P* values using Wald test. Finally, the linkage disequilibrium and haplotype construction were assessed using the Haploview software package (version 4.2) which developed and maintained by Dr. Mark Daly's lab at the MIT/Harvard Broad Institute, Massachusetts, United States and the SHEsis software platform (http://www.nhgg.org/analysis/).

## 3 Results

### 3.1 Study population

The distributions of sex and age among the cases and controls are shown in Table 1. A total of 285 cases (112 females and 173 males) and 308 controls (111 females and 197 males) were recruited for our study. The mean age of the patients was 41.88 ± 12.79 years and that of the controls was 49.47 ± 7.97 years. The mean age between patients and controls was not matched in present study, and we would adjust the factor in the followed analysis. The groups were well matched by sex (*P* = 0.413).

**Table 1:**
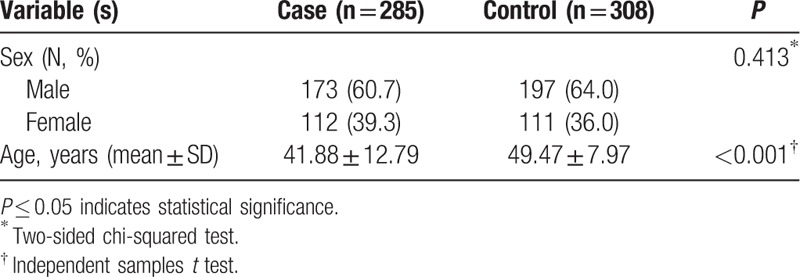
Characteristics of cases and controls in this study.

### 3.2 Hardy–Weinberg equilibrium and SNP alleles

The basic information about all the SNPs including gene, band, position, alleles, and HWE results are presented in Table 2. All of the 34 tag SNPs were in HWE among the control subjects (*P* > 0.05). Two SNPs were found to be significantly associated with an increased risk of steroid-induced ONFH (rs1940475T/C, OR = 1.32, 95% CI: 1.04–1.67; rs11225395A/G, OR = 1.32, 95% CI: 1.04–1.67).

**Table 2:**
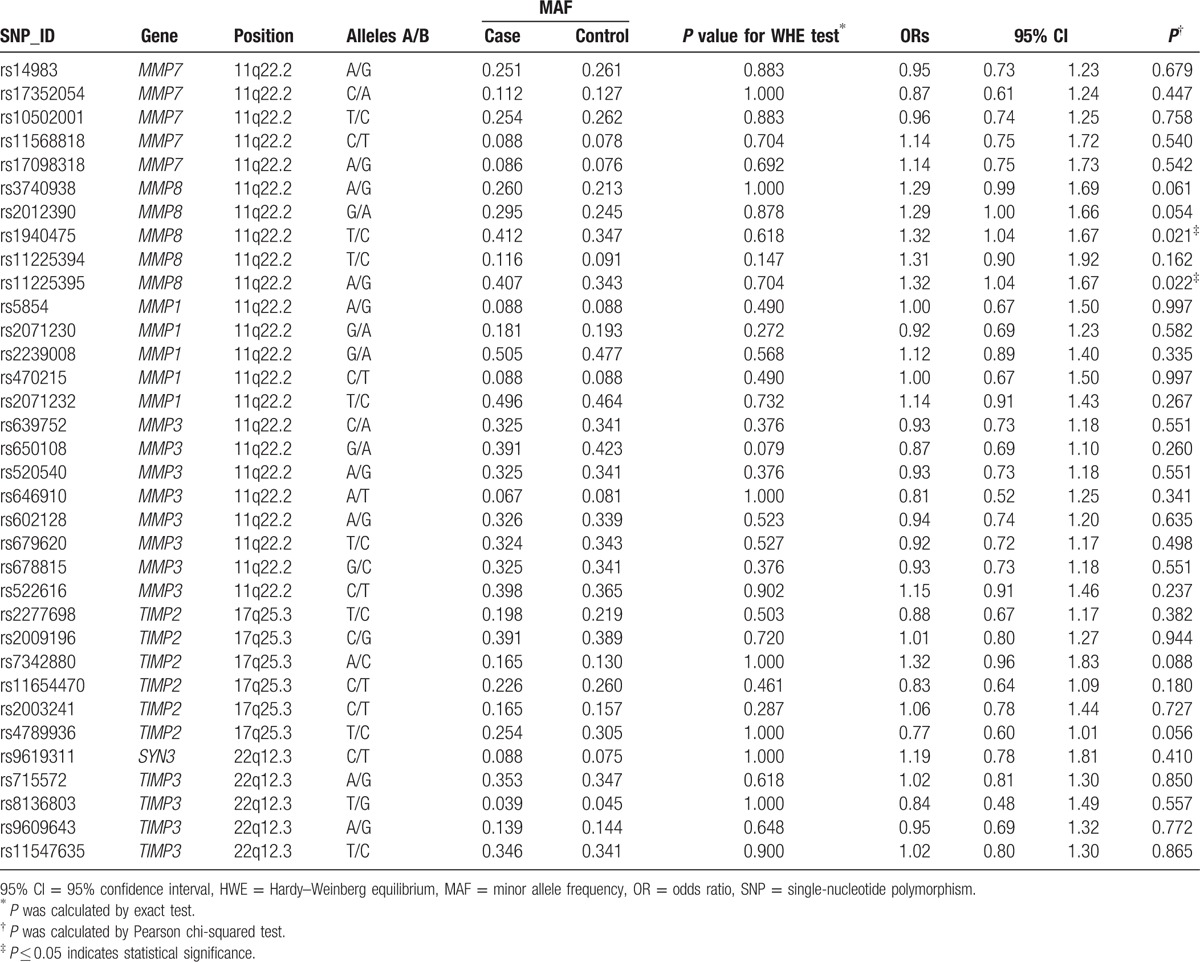
Allele frequencies in cases and controls and OR estimates for steroid-induced ONFH.

### 3.3 Association between MMP8 and the risk of steroid-induced ONFH

Genetic models (codominant, dominant, recessive, overdominant, and additive) and the genotype frequencies were used to further identify the associations between the SNPs and the risk of steroid-induced ONFH (Table 3). The results showed that rs1940475 significantly increased the risk of steroid-induced ONFH under the recessive model (OR = 1.67, 95% CI: 1.01–2.76, *P* = 0.042) and the additive model (OR = 1.36, 95% CI: 1.06–1.76, *P* = 0.016) and that rs11225395 exhibited an increased risk under both the recessive model (OR = 1.70, 95% CI: 1.03–2.81, *P* = 0.037) and the additive model (OR = 1.34, 95% CI: 1.04–1.73, *P* = 0.023). Meanwhile, rs3740938 showed a negative effect on steroid-induced ONFH under the dominant model (OR = 1.43, 95% CI: 1.01–2.04, *P* = 0.045) and the additive model (OR = 1.36, 95% CI: 1.02–1.82, *P* = 0.034). In addition, rs2012390 was found to be associated with an increased risk of steroid-induced ONFH under all of the genetic models including the codominant (OR = 1.56, 95% CI: 1.08–2.26, *P* = 0.046), dominant (OR = 1.55, 95% CI: 1.10–2.21, *P* = 0.013), overdominant (OR = 1.49, 95% CI: 1.04–2.13, *P* = 0.028), and additive (OR = 1.37, 95% CI: 1.04–1.82, *P* = 0.025) models. Collectively, these results, adjusted by age and gender, showed that 4 SNPs of *MMP8* (rs3740938, rs2012390, rs1940475, and rs11225395) had a negative effect on steroid-induced ONFH.

**Table 3:**
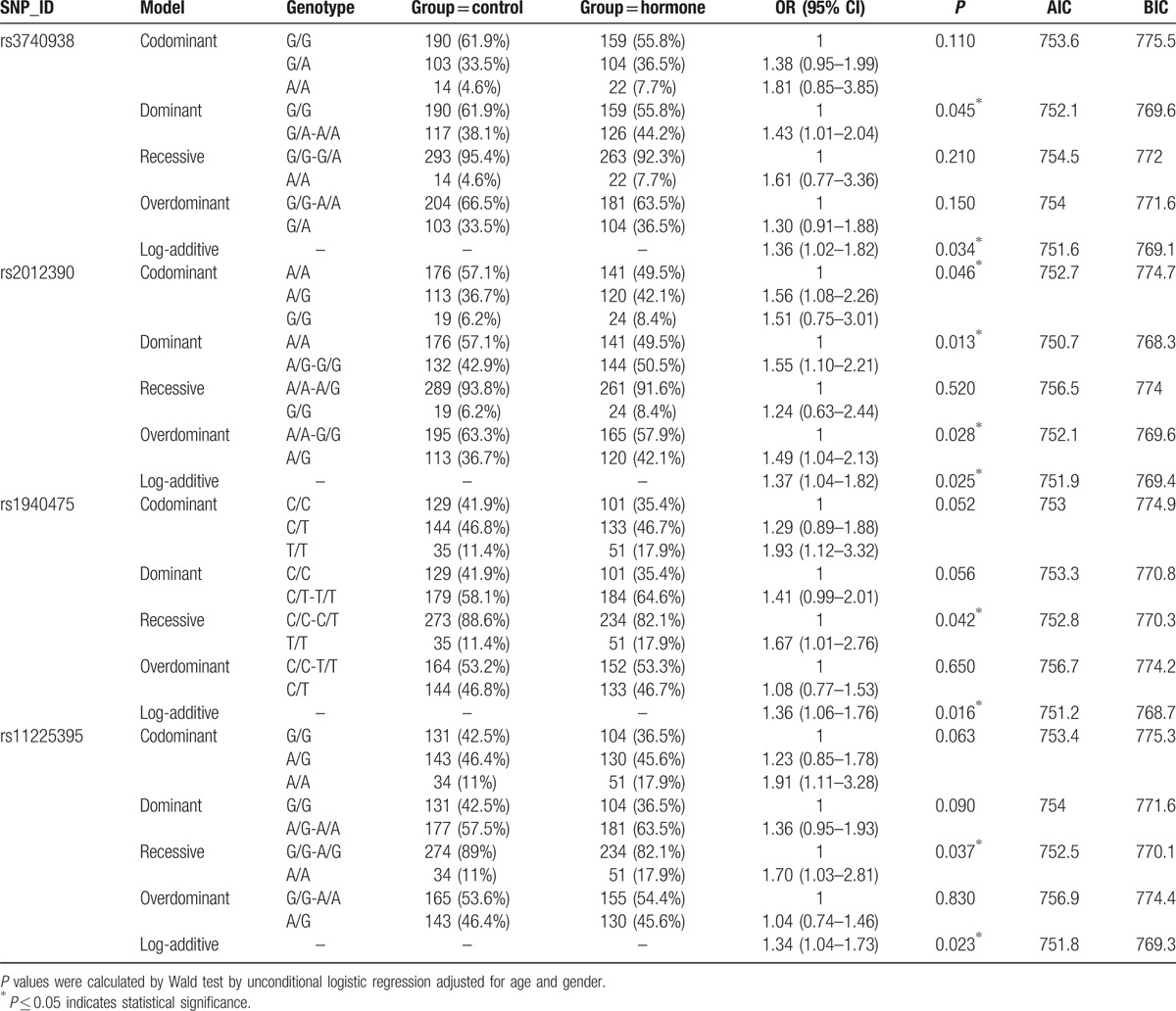
Genotypic model analysis of the relationship between SNPs and the risk of steroid-induced ONFH.

### 3.4 Stratified analysis of MMP8 polymorphisms by sex and the risk of steroid-induced ONFH

The results of the stratified analysis of the effects of rs3740938 and rs2012390 on steroid-induced ONFH are shown in Table 4. In the stratified analysis, there was no association between rs3740938 or rs2012390 and the risk of steroid-induced ONFH among males, whereas the associations between those SNPs and the risk of steroid-induced ONFH among females under the dominant model (rs3740938, OR = 2.69, 95% CI: 1.50–4.83, *P* = 0.001; rs2012390, OR = 2.30, 95% CI: 1.31–4.03, *P* = 0.012) and the additive model (rs3740938, OR = 2.02, 95% CI: 1.24–3.29, *P* = 0.010; rs2012390, OR = 1.77, 95% CI: 1.12–2.80, *P* = 0.047) were even stronger than those in the nonstratified analysis.

**Table 4:**
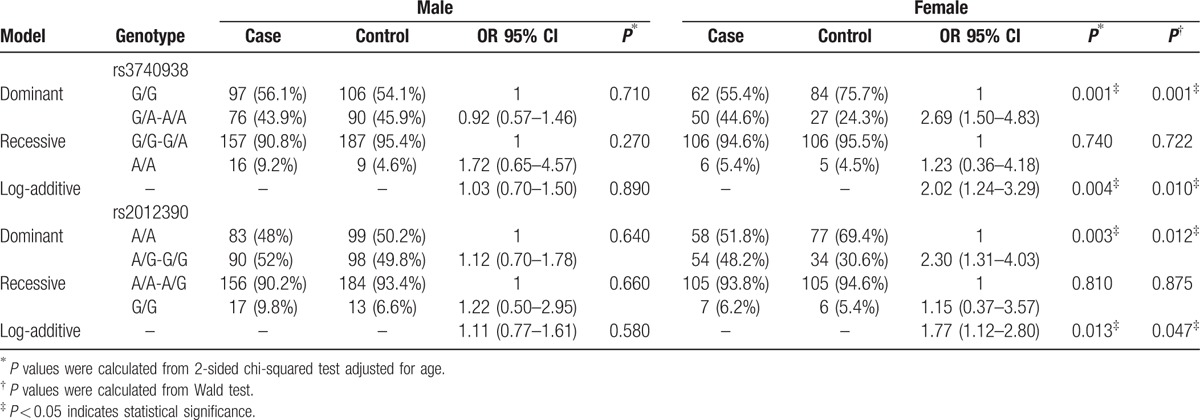
Stratified analysis of MMP8 polymorphisms by sex and risk of steroid-induced ONFH.

### 3.5 Association of MMP8 haplotypes with the risk of steroid-induced ONFH

**Figure 1:**
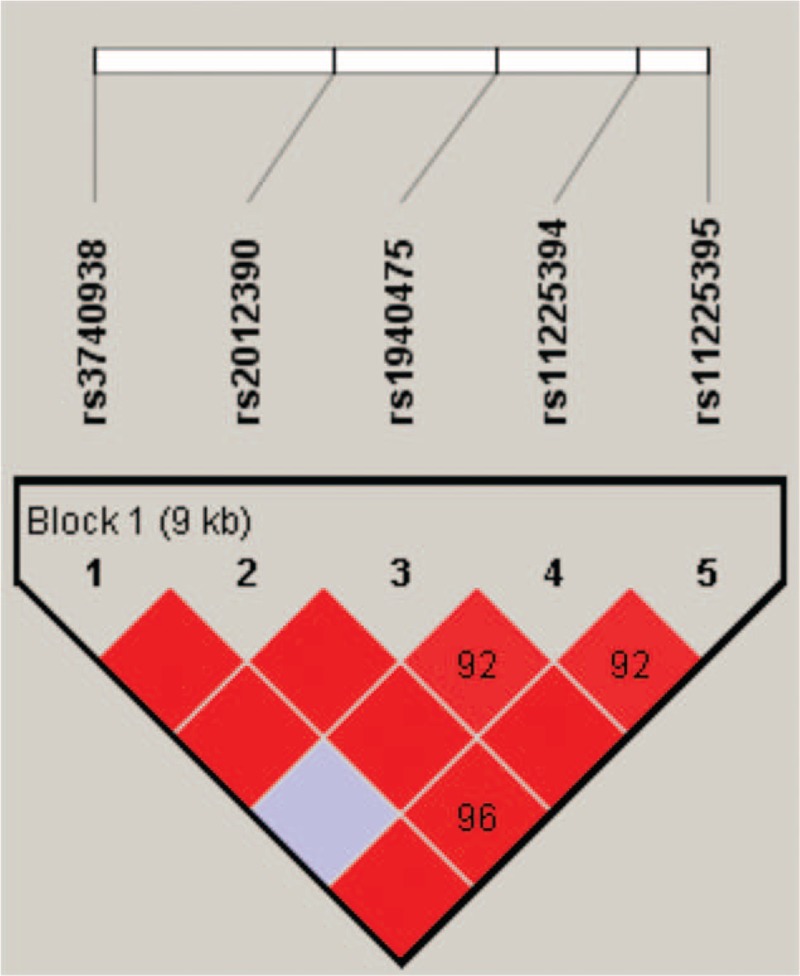
Haplotype block map for SNPs of the *MMP-8* gene. Linkage disequilibrium plots containing 5 SNPs from MMP-8. Red squares display statistically significant associations between a pair of SNPs, as measured by D′; darker shades of red indicate higher D′.

Finally, the linkage disequilibrium and haplotype construction were detected and evaluated. One block of *MMP8* SNPs (Fig. 1) comprising rs3740938, rs2012390, rs1940475, rs11225394, and rs11225395 was found by haplotype analysis. Compared with the “GACCG” wild-type, the “AGTCA” haplotype was found to be associated with an increased risk of steroid-induced ONFH (OR = 1.40, 95% CI: 1.04–1.88, *P* = 0.025; Table 5).

**Table 5:**
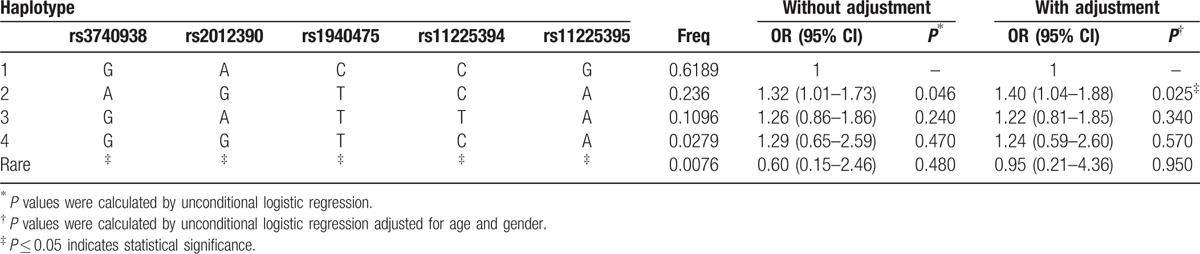
The haplotype frequencies of MMP8 polymorphisms and their association with the risk of steroid-induced ONFH.

## 4 Discussion

In the present case–control study, we investigated the associations between 34 SNPs of the MMP/TIMP system and the risk of steroid-induced ONFH. We demonstrated that *MMP8* genetic polymorphisms (rs3740938, rs2012390, rs1940475, and rs11225395) are associated with an increased risk of steroid-induced ONFH in the population of northern China. We found that gender differences might interact with *MMP8* polymorphisms to affect the development of steroid-induced ONFH. We also found that the “AGTCA” haplotype of *MMP8* was associated with a 1.40-fold increased risk of steroid-induced ONFH.

MMP-8, also known as collagenase-2 or neutrophil collagenase, can be expressed by a wide variety of cells such as maturing neutrophils, peripheral neutrophils, endothelial cells, and chondrocytes. MMP-8 plays an important role in a wide range of inflammatory disorders.^[27]^ MMP-8 initiates the first step of collagen degradation by disrupting triple helical fibrillar collagen.^[28]^ Billinghurst et al^[29]^ suggested that increased expression of MMP-8 would have an effect on the cleavage of type II collagen in human cartilage of osteoarthritis (OA). MMP-8 also possesses proteolytic activity on several matrix proteins, particularly some nonmatrix proteins such as angiotensin I. Fang et al^[30]^ reported that MMP-8 can cleave angiotensin I and convert it to angiotensin II, which in turn induces the endothelial cell expression of platelet or endothelial cell adhesion molecule-1 to promote angiogenesis. Hence, we suppose that polymorphisms of MMP-8 might have an effect on the inflammatory response or the circulatory impairment of the femoral head, leading further to steroid-induced ONFH.

Of the 4 SNPs in *MMP8* that were associated with increased risk of steroid-induced ONFH, rs3740938 and rs1940475 were located in exon coding regions; rs2012390 was located in an intron; and rs11225395 was located in a promoter region. It is possible that rs1940475 has an effect on proMMP-8 activation by a glutamate-to-lysine substitution at amino acid residue 87 located in the propeptide of MMP-8.^[31]^ The rs11225395 polymorphism has been shown by functional analysis to have an effect on MMP-8 promoter activity.^[31]^ The rs1940475 and rs11225395 polymorphisms have been reported to have an association with atherosclerosis. Ye^[32]^ conjectured that those SNPs might represent the same genetic signal, which arises from both the influence of rs11225395, located in the promoter region (position—799), on MMP-8 expression and the effect of rs1940475, located in the coding region (Glu87Lys), on MMP-8 zymogen activation, because the 2 SNPs were in strong linkage disequilibrium in Chinese and Japanese populations. In our study, rs1940475 and rs11225395 were found to have a significantly increased association with the risk of steroid-induced ONFH in both the allele model and the genetic models. We suppose that the rs1940475 and rs11225395 polymorphisms of *MMP8* might have a negative effect on the circulatory impairment of the femoral head as previous studies suggested that rs1940475 and rs11225395 have a functional role in MMP8 expression.^[33–35]^

Previously, a GWAS study conducted in Spain investigated the associations between rs3740938, rs2012390, and rs1940475 and OA, and rs1940475 showed a suggestive association with knee OA.^[36]^ In our study, rs3740938 and rs2012390 were associated with a significantly increased risk of steroid-induced ONFH under the genetic models. In addition, further stratification analysis showed that the associations between rs3740938 and rs2012390 and the risk of steroid-induced ONFH were significantly stronger in females than in males, which provides the first evidence of interactive effects between rs3740938 and rs2012390 and gender on the risk of steroid-induced ONFH.

To our knowledge, our study provides for the first time evidence of associations between the 4 SNPs (rs3740938, rs2012390, rs1940475, and rs11225395) of *MMP8* from the MMP/TIMP system and the risk of steroid-induced ONFH. Although there are important discoveries revealed in our study, our study has some limitations. First, our study does not include an analysis of biological functions, which will be crucial for elucidating the role of *MMP8* in steroid-induced ONFH. Second, steroid-induced ONFH can be classified into different clinical stages for further analysis. Third, the participants in our study were all Han Chinese individuals recruited from the Zhengzhou Traditional Chinese Medicine Traumatology Hospital, which might involve a selection bias. Fourth, we used a hospital-based case–control design, which may involve selection bias. Lastly, the sample size was relatively small after stratification by sex, which might convert the positive findings into negative results. A larger case–control study is expected to circumvent those problems, which could make our conclusions more powerful.

## 5 Conclusion

To sum up, we have confirmed for the first time that 4 susceptive SNPs (rs3740938, rs2012390, rs1940475, and rs11225395) of *MMP8* from the MMP/TIMP system exhibit a significant association with increased risk of steroid-induced ONFH in the population of northern China. Further functional studies and larger population-based studies are needed to confirm our results.

## Acknowledgments

It is our great honor to express heartfelt thanks to all of the patients and controls, the clinicians, and other hospital staff for their participation and contributions to this study.
